# Congenital nasal pyriform aperture stenosis with single central megaincisor

**DOI:** 10.31744/einstein_journal/2019AI4525

**Published:** 2019-02-25

**Authors:** Eduardo Kaiser Ururahy Nunes Fonseca, Rodrigo Watanabe Murakoshi, Rafael Maffei Loureiro, Daniel Vaccaro Sumi, Carolina Ribeiro Soares, Regina Lucia Elia Gomes, Mauro Miguel Daniel, Marcelo Buarque de Gusmão Funari

**Affiliations:** 1 Hospital Israelita Albert Einstein, São Paulo, SP, Brazil.

A 15-day-old female newborn presenting nasal obstruction since birth underwent computed tomography of the paranasal sinuses in our department due to clinical suspicion of choanal atresia. The patient presented no other complaints.

Computed tomography (Figure 1) allowed the diagnosis of pyriform aperture stenosis, which is a rare cause of nasal obstruction in neonates and was first described in 1989.^(^ ^1^ ^)^ Pyriform aperture stenosis becomes particularly significant during the first two months of life when infants are mandatory nose breathers.

**Figure 1:**
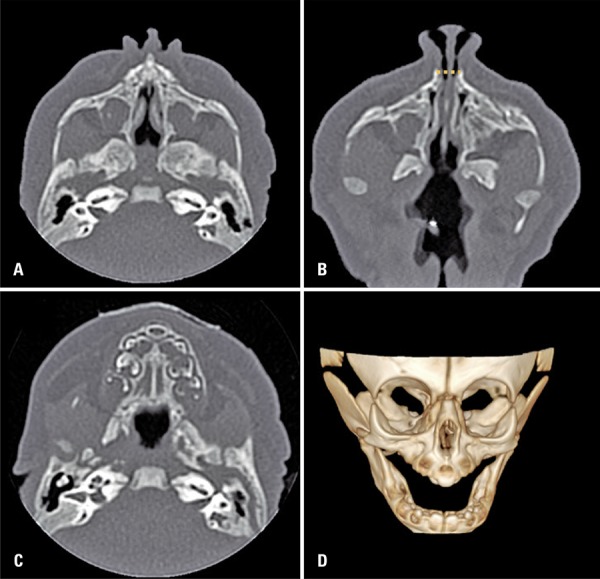
Axial computed tomography scan sections of paranasal sinuses. (A) Open choanae, no atresia. (B) Bilateral and symmetrical thickening and medialization of the anterior aspect of the maxilla, which determines extreme narrowing of pyriform aperture (dotted line, measuring about 0.6cm, normal value = 1.1cm). The image is compatible with congenital pyriform aperture stenosis. (C) Single central megaincisor in median maxillary position and hard palate with triangular configuration, both frequent findings in congenital pyriform aperture stenosis. (D) Three-dimension reconstruction shows narrowing of pyriform aperture and single central megaincisor

The lateral margins of the pyriform opening are defined by the nasal processes of the maxilla and its lower margins by the junction of the horizontal processes of the same bone.

Pyriform aperture stenosis is believed to be due to embryological malformations of the primary palate, which are associated with a hard triangular-like palate and the bone overgrowth of the nasal process of the maxilla. This set of findings narrows the pyriform aperture, which is particularly important since the pyriform aperture is one of the narrowest nose areas. Even small decreases in the pyriform aperture area cause significant increase in airway resistance.^(^ ^1^ ^-^ ^4^ ^)^

The clinical presentation is indistinguishable from choanal atresia, although it can be safely diagnosed by paranasal sinus computed tomography scan when the imaging reveals a diameter of the pyriform aperture below 1.1cm.^(^ ^2^ ^,^ ^5^ ^)^

Often there are additional malformations associated, mainly the triangular-shape palate and the single central megaincisor, which is found in up to 75% of these patients.^(^ ^2^ ^,^ ^5^ ^)^

The initial treatment of this condition is usually conservative. Surgical interventions are reserved for cases of persistent symptoms after conservative treatment.^(^ ^1^ ^,^ ^6^ ^)^

## References

[1] Brown OE, Myer CM, Manning SC (1989). Congenital nasal pyriform aperture stenosis. Laryngoscope.

[2] Rollins N, Booth T, Biavati M (2001). Case 40: congenital pyriform aperture stenosis. Radiology.

[3] Sesenna E, Leporati M, Brevi B, Oretti G, Ferri A (2012). Congenital nasal pyriform aperture stenosis: diagnosis and management. Ital J Pediatr.

[4] Thomas EM, Gibikote S, Panwar JS, Mathew J (2010). Congenital nasal pyriform aperture stenosis: a rare cause of nasal airway obstruction in a neonate. Indian J Radiol Imaging.

[5] Belden CJ, Mancuso AA, Schmalfuss IM (1999). CT features of congenital nasal piriform aperture stenosis: initial experience. Radiology.

[6] Visvanathan V, Wynne DM (2012). Congenital nasal pyriform aperture stenosis: a report of 10 cases and literature review. Int J Pediatr Otorhinolaryngol.

